# POU-M2 promotes juvenile hormone biosynthesis by directly activating the transcription of juvenile hormone synthetic enzyme genes in *Bombyx mori*

**DOI:** 10.1098/rsob.220031

**Published:** 2022-04-06

**Authors:** Rui Cai, Gang Tao, Ping Zhao, Qingyou Xia, Huawei He, Yejing Wang

**Affiliations:** ^1^ State Key Laboratory of Silkworm Genome Biology, Biological Science Research Center, Southwest University, Chongqing, People's Republic of China; ^2^ Chongqing Key Laboratory of Sericultural Science, Chongqing Engineering and Technology Research Center for Novel Silk Materials, Southwest University, Chongqing, People's Republic of China; ^3^ Chongqing Key Laboratory of Soft-Matter Material Chemistry and Function Manufacturing, Southwest University, Chongqing, People's Republic of China

**Keywords:** *Bombyx mori*, POU-M2, juvenile hormone biosynthesis, JHAMT, transcriptional regulation

## Abstract

Juvenile hormone (JH) plays a key role in preventing larval precocious metamorphosis, maintaining larval state, controlling adult sexual development and promoting insect egg maturation. Genetic studies have shown that POU factor ventral veins lacking regulates JH synthesis to control the timing of insect metamorphosis. However, how POU factor regulates JH synthesis is largely unknown. Here, we found POU-M2 was highly expressed in corpora allata (CA) and specifically localized in the nucleus of CA. The overexpression of POU-M2 promoted the expression of JH synthase genes and kr-h1 and enhanced the activity of JH synthase genes promoter. Further, POU-M2 promoted the transcription of JH acid O-methyltransferase (JHAMT) by directly binding to the key cis-regulatory elements -207, -249 and -453 within the proximal regions of JHAMT promoter. Both the POU domain and homeodomain were vital for the activation of POU-M2 on JHAMT transcription. Our study reveals the mechanism by which POU-M2 regulates JHAMT transcription.

## Graphical abstract

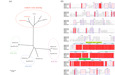

## 1. Introduction

Many animals including amphibians, invertebrates, vertebrates and insects undergo tremendous morphological changes from immature larvae to mature adults. The dramatic transformations are mediated by endocrine hormones during post-embryonic development. The main regulatory steroids are rogens and oestrogens in males and females of mammals, respectively [1]. 20-Hydroxyecdysone (20E) and juvenile hormone (JH) are two major endocrine hormones that synergistically regulate the developmental transition of insects [2,3]. 20E is synthesized in the prothoracic gland (PG), and JH is secreted by a pair of corpora allata (CA) located on either side of the brain. JH prevents larvae from moulting and pupating when JH is present at a relatively high level in the larval stage; whereas while JH decreases to very low levels or is absent in the last instar, 20E induces larval–larval moulting and larval–pupal–adult metamorphosis [4–8]. Thus, JH plays a key role in preventing 20E-induced precocious metamorphosis of larvae into pupae and adults [9–12].

The POU gene family is ubiquitous in vertebrates and invertebrates, and plays vital roles in cell type-specific gene expression and cell fate determination [13]. POU factors contain a highly conserved homeodomain and a POU-specific domain. The POU-specific domain determines the high-affinity and site-specific DNA-binding capacity of POU factor [14,15]. POU factors affect the development of vertebrates' neuroendocrine system during the juvenile stage and puberty [16–20]. In *Drosophila melanogaster*, the POU factor drifter regulates cell proliferation and differentiation of wing imaginal discs [21], the differentiation and migration of tracheal cells and neurons [22,23], and the expression of dopa decarboxylase [24]. In *Bombyx mori*, the POU factor POU-M1 is involved in the transcription of sericin-1 gene [25], and POU-M2 regulates the expression of fibroin heavy chain (fib-H) [26,27], wing disc cuticle protein 4 (WCP4) [28,29], steroidogenic enzyme phantom [30], vitellogenin (Vg) [31], vitellogenin receptor (VgR) [32], and diapause hormone and pheromone biosynthesis-activating neuropeptide (DH-PBAN) [33]. Also, POU-M2 regulates the expression of DH-PBAN and phosphatase and tensin homologue in *Helicoverpa armigera* [34,35]. Recent studies show silencing of ventral veins lacking (vvl) by RNA interference (RNAi), a homologue of POU-M2, results in early metamorphosis and reduced the expression of JH acid methyltransferase 3 (JHAMT3) in *Tribolium castaneum* [36], and JH response gene Krüppel homologue 1 (kr-h1) in the fat body of *Oncopeltus fasciatus* during reproduction [37], implying the crucial role of vvl in JH biosynthesis. However, the role of POU-M2 in JH biosynthesis remains largely unknown in *Bombyx mori*.

Here, we found that POU-M2 was highly expressed in CA and specifically localized in the nucleus of CA cells of the silkworm. The expression of POU-M2 was similar to that of JH synthetic enzyme genes from the third larval instar (L3) to the third day of the fifth larval instar (L5D3). The overexpression of POU-M2 promoted the expression of JH synthetic enzyme genes and kr-h1. In particular, POU-M2 promoted the transcription of JH synthetic key enzyme JH acid O-methyltransferase (JHAMT) in a dose-dependent manner. Electrophoretic mobility shift assay (EMSA) suggested POU-M2 directly bound to the key cis-regulatory elements (CREs) within the proximal region of JHAMT promoter, which was further verified by chromatin immunoprecipitation (ChIP)-PCR in cells and in the CA of silkworm larvae. Both POU the domain and homeodomain were essential for the activation of POU-M2 on JHAMT promoter. Our study suggests that POU-M2 promotes JH biosynthesis by directly activating the transcription of JH biosynthetic enzyme genes in *B. mori*.

## 2. Material and methods

### 2.1. Insects and cell lines

*Bombyx mori* strain Dazao was provided by the State Key Laboratory of Silkworm Genome Biology, Southwest University, Chongqing, China. Silkworm larvae were reared on fresh mulberry leaves under a 12 L : 12 D photoperiod at 25°C and 75% relative humidity. The silkworm embryo-derived (BmE) cells are widely used in the study of the silkworm [28,30]. BmE cells were cultured in Grace's medium (Gibco, MA, USA) containing 10% fetal bovine serum (Hyclone, Logan, UT, USA) and standard cell culture antibiotics (Gibco) at 27°C.

### 2.2. Quantitative real-time PCR

Total RNA was extracted from brain–corpora cardiaca–corpora allata (Br-CC-CA) and corpora cardiaca–corpora allata (CC-CA) using PureLink RNA microkit (Invitrogen, MA, USA), and from BmE cells using Total RNA kit II (Omega, GA, USA), respectively. cDNA was synthesized using oligo(dT) and reverse transcriptase kit (M-MLV) (Promega, WI, USA) with 1 µg total RNA as the template. Quantitative real-time (qRT)-PCR was performed using SYBR Premix Ex Taq kit (Takara, Japan) on 7500 Fast RT-PCR System (Applied Biosystems, USA) in 20 µl reaction mixture containing 10 μl SYBR Premix Ex Taq (2×) and 0.8 µl each primer (10 mM). The program was set as initial denaturation at 95°C for 30 s, 40 cycles (Cq) at 95°C for 5 s and 60°C for 30 s. Glyceraldehyde 3-phosphate dehydrogenase (GAPDH) was used as an internal reference. The primers for qRT-PCR are listed in the electronic supplementary material, table S1. Relative mRNA level was calculated using the 2^−ΔΔCT^ method [38]. All experiments were conducted with three independent biological replicates.

### 2.3. Vector construction and cell transfection

The open reading frame of POU-M2 was subcloned into a modified pSLfa1180fa vector with insertion of hr3 enhancer into the proximal region of BmAct4 promoter for overexpression. POU-M2^ΔP^, POU-M2^ΔH^ and POU-M2^ΔPH^ were POU-M2 variants with the deletion of POU, homeodomain and both domains, respectively. These variants fragments were cloned and inserted into a modified pSLfa1180 vector pSL1180 [A4-X-SV40], where X was the target DNA fragments. The coding region of enhanced red fluorescent protein (DsRed) was cloned into the same vector as a control. The accession number of acetoacetyl-CoA thiolase (*AACT*), HMG-CoA synthase (*HMGS*), HMG-CoA reductase (*HMGR*), diphosphomevalonate decarboxylase (*MevPPD*), farnesyl diphosphate synthase 2 (*FPPS2*), mevalonate kinase (*MevK*) and *JHAMT* genes in PubMed database are 100 101 202, 100 101 203, 100 101 204, 100 101 206, 100 101 207, 100 101 205 and 692445, respectively. The −2220 ∼ +1 bp upstream of AACT promoter, the −1947 ∼ +1 bp upstream of HMGS promoter, the −2075 ∼ +1 bp upstream of HMGR promoter, the −2042 ∼ +1 bp upstream of MevPPD promoter, the −2279 ∼ +1 bp upstream of FPPS2 promoter, the −1985 ∼ +1 bp upstream of MevK promoter and the −2501 ∼ +1 bp upstream of JHAMT promoter were generated by PCR using Dazao genome as the template and then inserted into pGL3-basic vector, respectively. All primers for the cloning of JH synthase genes are shown in electronic supplementary material, table S1. Different 5′-truncated promoters of JHAMT were generated by PCR and then inserted into a pGL3-basic vector. The core-binding elements AT of the key CREs−207, −249 and/or −453 were all mutated to CG by targeted mutagenesis. The pGL3-JHAMT vector was cotransfected into BmE cells with Renilla luciferase reporter vectors using X-treme GENE HP DNA transfection reagent (Roche, Swiss). Luciferase activity was measured using the Duai-Glo luciferase assay system (Promega). Each transfection was repeated three times independently (*n* = 3).

### 2.4. Western blot

BmE cells were incubated with RIPA lysis buffer (Beyotime, Beijing, China) containing protease inhibitor cocktail (Sigma, USA) for 30 min and then centrifuged at 12 000*g* at 4°C for 15 min. The supernatant (10 µg protein/sample) was separated on 12% sodium dodecyl sulfate-polyacrylamide gels by electrophoresis (SDS-PAGE) and then transferred to polyvinylidene difluoride membrane (GE Healthcare, USA). The membrane was blocked in TBST buffer (10 mM Tris-HCl, 150 mM NaCl, 0.05% Tween 20 (v/v), pH 7.5) containing 5% skim milk (w/v) at 37°C for 1 h and then incubated with anti-POU-M2 at 4°C overnight. Anti-POU-M2 was diluted as a ratio of 1 : 10 000 in TBST buffer containing 1% skim milk (w/v). After washing with phosphate-buffered saline (PBS, pH 7.0) thrice, the membrane was incubated with horseradish peroxidase-conjugated goat anti-rabbit IgG (dilution ratio, 1 : 20 000) (Dingguo Biotech., Nanjing, China) at 37°C for 2 h. The signal was observed on the SH-523 chemiluminescence imaging system (SHST, Hangzhou, China) using the supersignal west femto maximum sensitivity substrate (Thermo Fisher, MA, USA).

### 2.5. Immunostaining

Br-CC-CA was fixed in 4% paraformaldehyde at 25°C for 30 min, washed thrice with 0.3% PBST (PBS containing 0.3% Triton-X 100 (v/v)) and then incubated with anti-POU-M2 (dilution ratio: 1 : 1,000) at 4°C overnight. Next, the samples were incubated with Cy3-labelled goat anti-rabbit IgG (dilution ratio: 1 : 500) (Beyotime) for 2 h followed by washing with PBS thrice and cell nuclei staining with 4′,6-diamidino-2-phenylindole (dilution ratio, 1 : 1000) (Life Technologies, CA, USA). Finally, the samples were fixed in an anti-fade medium (Beyotime) after washing with PBS buffer thrice and then imaged on an Olympus confocal microscopy FV1000 (Tokyo, Japan). The fluorescence signal was excited at the wavelength of 340 nm and 550 nm, respectively.

### 2.6. Electrophoretic mobility shift assay

DNA oligonucleotides labelled with biotin at the 5′-end were annealed to produce double-stranded probes. After the overexpression of POU-M2 in BmE cells, the nuclear proteins were extracted from the cells using the nuclear and cytoplasmic protein extraction kit (Beyotime). EMSA was performed using an EMSA/Gel-Shift kit (Beyotime) [39]. DNA-binding assay was performed in 10 µl reaction system containing 2 µl nucleoprotein, 1 µl labelled probe and 2 µl binding buffer (Beyotime) at 25°C for 20 min. For the competition assay, a 10- to 100-fold molar excess of unlabelled (cold) probe or mutant probe was incubated with nucleoprotein for 10 min and then incubated with the labelled probe for 20 min. For antibody-based EMSA analysis, the nucleoprotein, labelled probe and anti-POU-M2 (1 µl) were co-incubated at 25°C for 20 min. The reaction mixture was separated on 5% SDS-PAGE in 0.5 × TBE buffer (45 mM Tris-borate, 1 mM EDTA, pH 8.3) by electrophoresis. Finally, the gel was photographed by a Bio-Rad Typhoon scanner (CA, USA). The primers for the probes are listed in the electronic supplementary material, table S1.

### 2.7. Chromatin immunoprecipitation-PCR

POU-M2 was overexpressed in BmE cells. CC-CA was isolated from silkworm larvae on day 1 of the fourth larval instar (L4D1). BmE cells and CC-CA were immobilized with 37% formaldehyde to cross-link with the chromatin and then sheared into 200–1000 bp DNA fragments by sonication. ChIP was carried out using EZChIP kit (Millipore, MA, USA). Genomic PCR analysis was performed using specific primers covering the proximal CREs of JHAMT promoter. Anti-IgG and anti-RNA polymerase II (anti-PolyII) were used as negative and positive controls, respectively. For immunoprecipitation, protein–DNA conjugates were enriched with 1 μg rabbit anti-IgG, anti-POU-M2 and anti-PolyII, respectively. DNA fragments were isolated from the immunoprecipitates for PCR amplification using primers in the electronic supplementary material, table S1. PCR products were separated by 2% agarose gel and then identified by DNA sequencing.

### 2.8. Statistics

The data were reported as the mean of at least three independent tests ± standard deviation and analysed using Student's *t*-test and variance analysis, respectively. For *t*-test: ***, *p* < 0.001; **, *p* < 0.01; *, *p* < 0.05; n.s., no significant difference.

## 3. Results

### 3.1. Homology analysis of POU-M2

**Figure 1.**
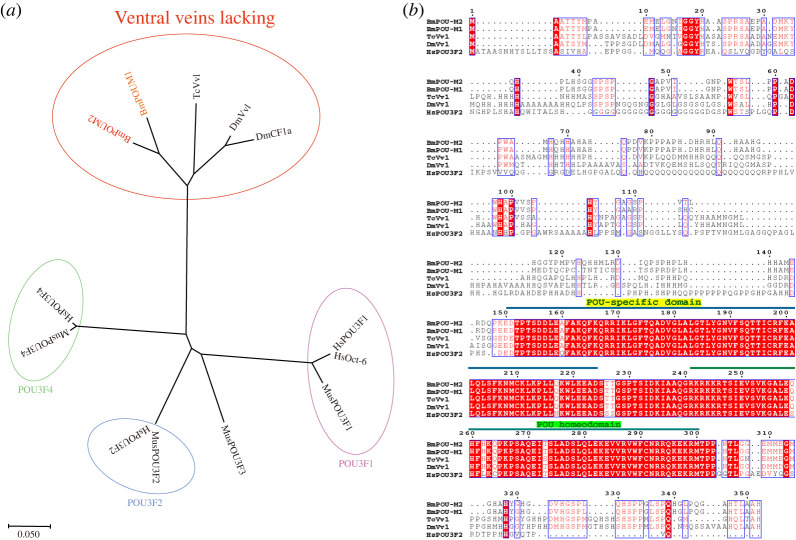
Phylogenetic analysis and sequence alignment. (*a*) Phylogenetic analysis. The accession numbers are as follows: BmPOU-M2 (NP_001037456.2); BmPOU-M1 (Q17237); TcVvl (NP_001139385.1); DmVvl (NP_001286957.1); DmCF1a (CAA36496.1); MusPOU3F1 (EDL30360.1); MusPOU3F2 (NP_032925.1); MusPOU3F3 (NP_032926.2); MusPOU3F4 (NP_032927.1); HsOct-6 (CAA 79158.1); HsPOU3F1 (NP_002690.3); HsPOU3F2 (NP_005595.2); HsPOU3F4 (NP_000 298.3). (*b*) Sequence alignment of POU factors. Conserved amino acids are highlighted in red shadow.

To analyse the homology of POU-M2 in different species, an evolutionary tree was constructed for phylogenetic analysis (figure 1*a*) using POU-M2 homologues from *B. mori*, *D. melanogaster*, *T. castaneum*, *Mus musculus* and *Homo sapiens*. The results showed that POU-M1 and POU-M2 were in a subgroup that clustered with Dmvvl and Tcvvl (figure 1*a*), implying that they may have evolutionarily similar origins. Other homologues were clustered in different subgroups according to their evolutionary proximity (figure 1*a*), respectively.

Sequence alignment was performed using ClustalX [40] to determine the conserved domains of POU factors in different species. The results showed that the POU-specific domain and POU-homeodomain (highlighted in red shadow) were highly conserved across species (figure 1*b*), implying POU-M2 may act similarly to its homologue vvl.

### 3.2. POU-M2 is localized in the nucleus of corpora allata cells

**Figure 2.**
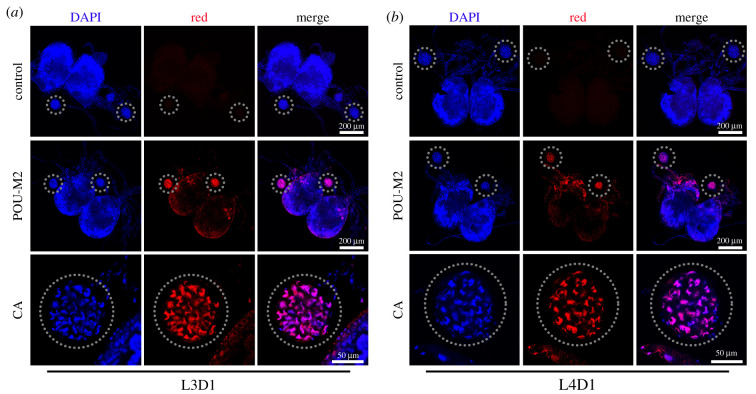
Immunostaining of Br-CC-CA. Br-CC-CA was dissected from silkworm larvae (L3D1) (*a*) and (L4D1) (*b*), then incubated with anti-POU-M2. PBS was used as a control. The grey dashed circles indicated the location of CA.

The expression profile showed that POU-M2 was highly expressed in Br-CC-CA, anterior silk gland and middle silk gland relative to other tissues of silkworm larvae (L5D3) (electronic supplementary material, figure S1), implying a key role of POU-M2 in these tissues. Further, immunostaining indicated that POU-M2 was specifically located in the nucleus of CA cells of silkworm larvae on day 1 of the third/fourth instar (L3D1/L4D1) (figure 2), implying POU-M2 may be involved in JH biosynthesis in Br-CC-CA.

### 3.3. Temporal expression profiles of POU-M2 and juvenile hormone synthase

**Figure 3.**
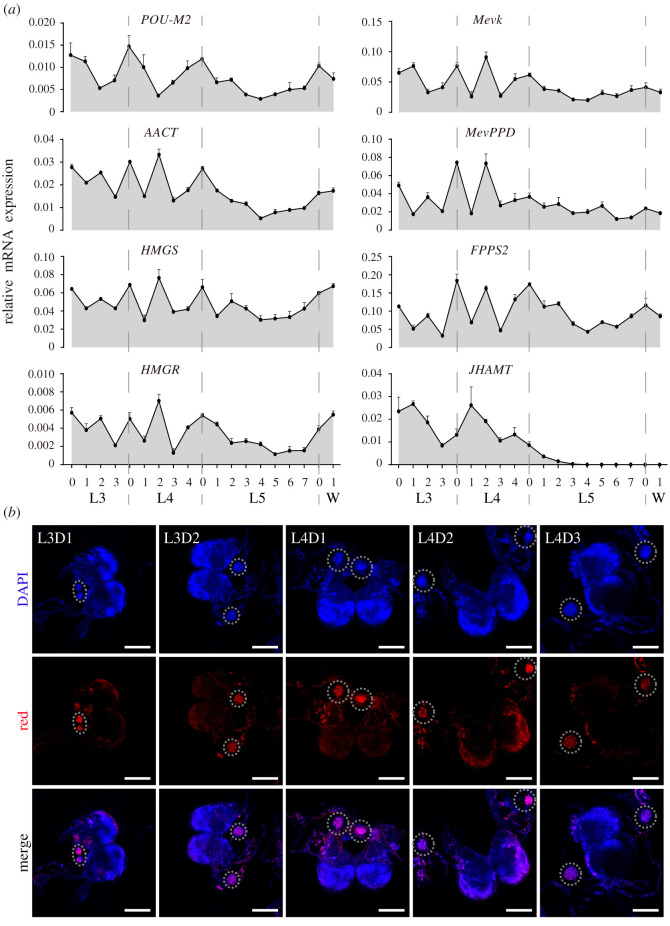
Temporal expression profiles of POU-M2 and JH synthase in CA. (*a*) qRT-PCR of POU-M2 and JH synthase genes from L3 to day 1 of the wandering stage (W1). GAPDH was used as a reference. The vertical dotted lines indicated the timing of each stage. (*b*) Immunostaining of Br-CC-CA from L3 to L4 using anti-POU-M2. Scale bar, 200 μm.

To reveal the effect of POU-M2 on JH synthase expression, we compared the expression profiles of POU-M2 and JH synthase genes in CA at different developmental stages. The results showed POU-M2 showed relatively high expression at the beginning and end of each instar, and low at the middle of each instar, which was generally similar to the expression profiles of JH synthase genes. From the third instar (L3) to the fourth instar (L4), JHAMT expression was similar to that of POU-M2, but with a 24 h delay. JHAMT expression gradually decreased to a trace level from L4D4 to L5D3 and then was completely turned off (figure 3*a*). Immunostaining showed that the expression of POU-M2 at protein level in CA was consistent with that of POU-M2 at mRNA level from L3 to L4 (figure 3*b*). The results implied POU-M2 may be associated with the expression of JH synthase genes, especially JHAMT in the CA of silkworm larvae.

### 3.4. POU-M2 promotes the transcription of juvenile hormone synthase genes

**Figure 4.**
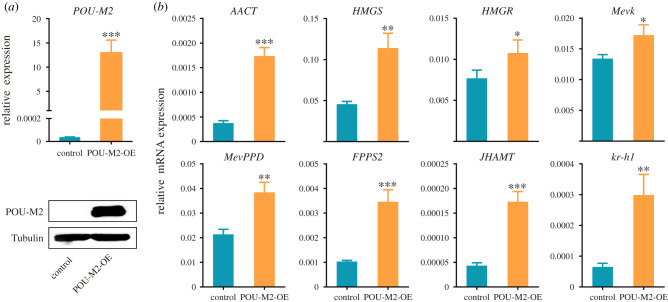
Overexpression of POU-M2 promoted the transcription of JH synthase and kr-h1. (*a*) qRT-PCR and western blotting analysis of POU-M2 overexpression in BmE cells. dsRed was used as a control. (*b*) qRT-PCR of JH synthase and kr-h1. OE, overexpression.

To reveal the relation of POU-M2 and JH synthase genes, the effect of POU-M2 on the expression and promoter activity of JH synthase genes were analysed after the overexpression of POU-M2 in BmE cells. qRT-PCR and western blotting confirmed the successful overexpression of POU-M2 (figure 4*a*). qRT-PCR showed the transcription levels were upregulated by about threefold for AACT, FPPS2, JHAMT and kr-h1, twofold for MevPPD and HMGS (figure 4*b*), respectively, suggesting that overexpression of POU-M2 promoted the transcription of JH synthase and kr-h1.

**Figure 5.**
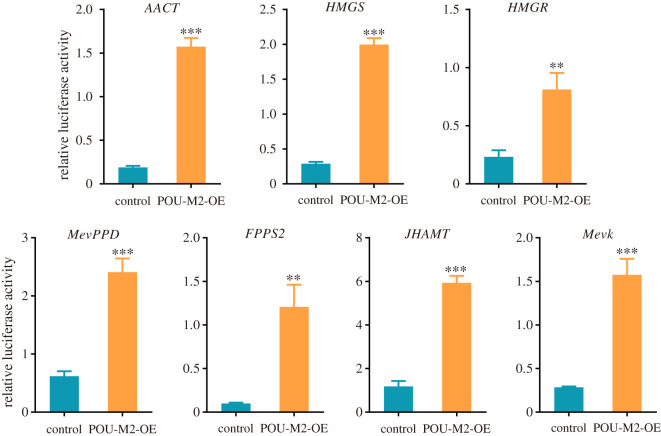
Luciferase reporter assay of the promoter activity of JH synthetic enzyme genes after the overexpression of POU-M2. OE, overexpression.

Since the sequence identity of the conserved domains between POU-M2 and Dmvvl is about 98%, we used JASPAR (https://jaspar.genereg.net/) to predict the potential CREs of POU-M2 upstream of JHAMT promoter. Jaspar is a high-quality transcription factor-binding profiles database. The top-scoring CREs were chosen as potential candidates for further validation using luciferase reporter and EMSA assays. The 2.5 kb sequence upstream of JH synthase genes was predicted to contain potential CREs binding to POU-M2. Dual-luciferase reporter assay showed the overexpression of POU-M2 activated the activity of JH synthase genes promoter (figure 5), indicating POU-M2 directly promotes the transcription of JH synthetic enzyme genes.

### 3.5. POU-M2 activates juvenile hormone acid O-methyltransferase promoter activity

**Figure 6.**
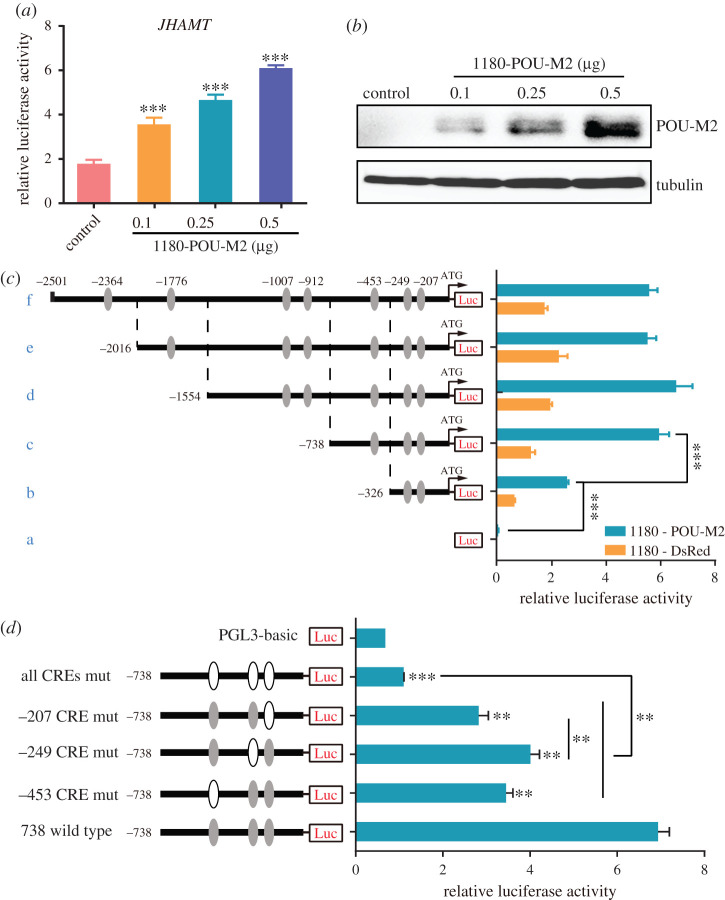
POU-M2 activated JHAMT promoter in BmE cells. (*a*) Relative luciferase activity of JHAMT promoter after transfection with different amounts of POU-M2. (*b*) Western blotting analysis of POU-M2 overexpression. (*c*) Relative luciferase activity of different JHAMT promoters. dsRed was used as the control. (*d*) Relative luciferase activity of JHAMT promoter mutants.

JHAMT is a key rate-limiting enzyme for JH biosynthesis [41]. Dual-luciferase reporter assay showed that the luciferase activity increased with the increase of POU-M2 (figure 6*a,b*), suggesting POU-M2 activates JHAMT promoter in a dose-dependent manner. Further, different 5′-flanking truncated promoters were generated to determine the key CREs for JHAMT promoter activity. There were no significant differences among promoters (c–f). The activity of promoter (b) was higher than that of promoter (a), but lower than that of promoter (c) (figure 6*c*), suggesting promoter (c) contained the key CREs for JHAMT promoter activity.

To reveal the role of the identified key CREs on the promoter activity, we carried out the reporter assay with constructs containing site-specific mutations in the key CREs-207, -349 and -453 of promoter (c). The core-binding motif AT of -207, -349 and/or -453 were all mutated to CG. The results showed the promoter activity was about half of the wild-type when the core-binding sites AT were mutated to CG, respectively, and was reduced to the lowest when all CREs were mutated simultaneously (figure 6*d*). The results suggested that the CREs-207, -249 and -453 in the promoter (c) are critical for the transcriptional activation of POU-M2 on JHAMT promoter.

### 3.6. POU-M2 directly binds to the key cis-regulatory elements of juvenile hormone acid O-methyltransferase promoter

**Figure 7.**
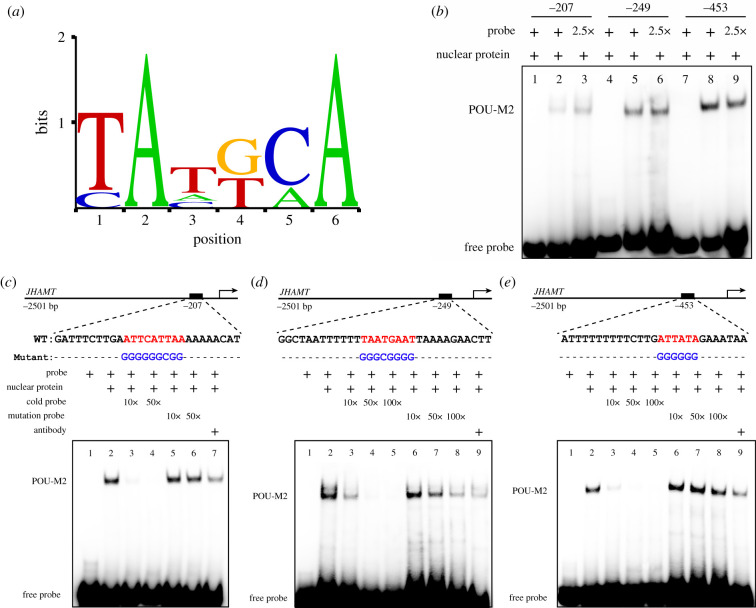
Analysis of the binding of POU-M2 to the key CREs upstream of JHAMT promoter. (*a*) Weblogo analysis of POU-M2-binding motifs. (*b*–*e*) EMSA analysis of the binding of POU-M2 to CREs. The probe sequences are shown at the top of each gel (*c*–*e*). Red and blue letters represented the core and mutated-binding motifs for POU-M2, respectively.

Weblogo [42] showed POU-M2 prefers to bind to AT-riched motifs (figure 7*a*). EMSA indicated that the nuclear protein from BmE cells overexpressing POU-M2 bound to the 5′-biotin-labelled oligonucleotide probes (figure 7*b–e*, lane 2), and this binding was competitively repressed by the unlabelled/cold probes (figure 7*c*, lanes 3–4; figure 7*d,e*, lanes 3–5). Further, the unlabelled mutant probes did not suppress (figure 7*c*, lanes 5–6; figure 7*e*, lanes 6–8) or partially repressed the binding of nucleoprotein to the labelled probes (figure 7*d*, lanes 6–8). Although the specific super-shift band did not appear, anti-POU-M2 repressed the binding of nuclear proteins to the labelled probes (figure 7*c*, lane 7; figure 7*d,e*, lane 9). The results suggested that POU-M2 directly binds to the key CREs -207, -249 and -453 upstream of JHAMT promoter.

### 3.7. Chromatin immunoprecipitation-PCR validation of the key cis-regulatory elements

**Figure 8.**
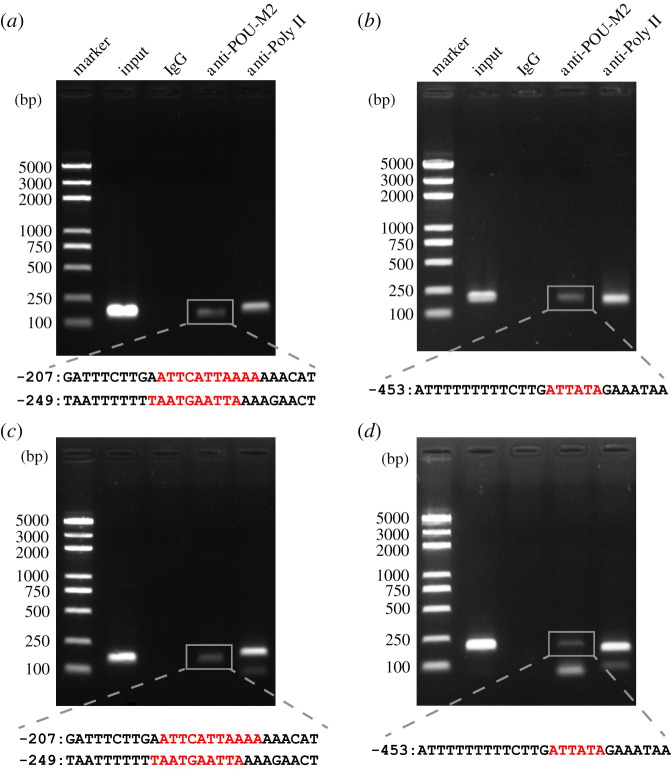
ChIP-PCR verification of the binding of POU-M2 to the key CREs in BmE cells (*a*,*b*) and in the CA of silkworm larvae (L4D1) (*c*,*d*). Red letters represent the core motifs for POU-M2 binding to the CREs. IgG and anti-Poly II were used as negative and positive controls, respectively.

To further validate the binding of POU-M2 to the key CREs, ChIP-PCR was performed after overexpression of myc-tagged POU-M2 in BmE cells. PCR showed the specific bands from anti-POU-M2 group were consistent with those from input DNA. DNA sequencing indicated the bands contained -207, -249 and -453 CREs of JHAMT promoter, but no bands appeared in the control IgG group (figure 8*a,b*), indicating the specific binding of POU-M2 to the key CREs in BmE cells. Further, the specific CREs upstream of JHAMT promoter were also identified in the CA of silkworm larvae (L4D1) (figure 8*c,d*). The results confirmed the binding of POU-M2 to the key CREs-207, -249 and -453 are present and specific *in vivo*.

### 3.8. POU domain and homeodomain are vital for POU-M2 activation

**Figure 9.**
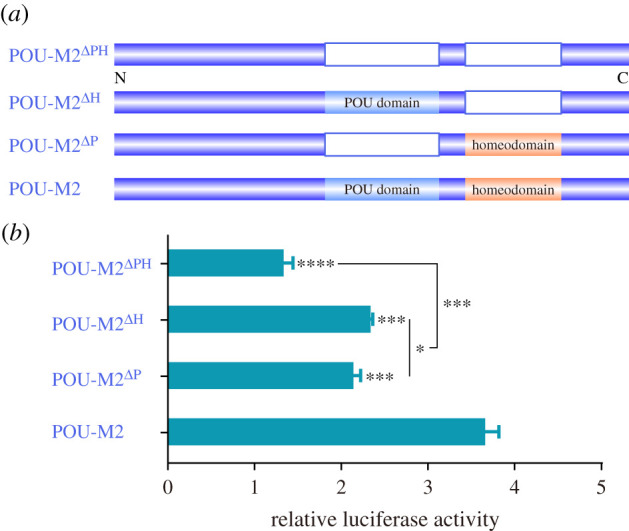
Identification of vital domains for the activation of POU-M2 on full-length JHAMT promoter (−2501 bp). (*a*) An illustration of POU-M2 mutants with different domains deletion. (*b*) Relative luciferase activity of JHAMT promoter (−2501 bp) after overexpression of POU-M2 mutants and POU-M2.

To identify the key domain for POU-M2 activation, POU-M2 mutants with different domains deletion were generated (figure 9*a*) and then overexpressed in BmE cells to compare the difference of the luciferase activity of full-length JHAMT promoter (-2501 bp). The results showed the luciferase activity of POU-M2 mutants were lower than that of POU-M2 (figure 9*b*), suggesting both the POU domain and homeodomain are crucial for the activation of POU-M2 on JHAMT promoter.

## 4. Discussion

The developmental transition from immature larvae to reproductive adults in insects is primarily regulated by ecdysone and JH [2,3,43]. Although the signalling network of hormones biosynthesis [44,45] and their signalling pathway [46,47] have been elucidated for a long time, little is known about the transcriptional regulation of JH biosynthesis.

It is known that the embryonic growth and morphogenesis of silkworm are independent of JH. JH is not considered a key factor in the development of transgenic silkworms overexpressing JH esterase from embryo to the second larval instar [8]. Precocious metamorphosis occurs after the second larval instar in the absence of JH or JH signalling [48]. Although JH is detected during the embryonic stage, JH signalling is not activated until the third instar. Therefore, the third and fourth instar larvae were chosen to study the regulation of POU-M2 on JH biosynthesis in this study.

Recently, Cheng *et al*. have found that RNAi of vvl reduces the expression of JHAMT3, ecdysone response gene hormone receptor 3 (HR3), ecdysone synthesis gene phantom and spook, and causes precocious metamorphosis and impaired moulting in *T. castaneum* [36], suggesting that vvl may affect JH and ecdysone synthesis. Sarwar *et al*. find silencing of vvl by RNAi reduces the expression of HR3, spook and kr-h1, and impacts JH and ecdysteroid synthesis in *O. fasciatus*, indicating vvl may be a key factor in JH and ecdysteroid synthesis [37]. However, how vvl regulates JH biosynthesis remains poorly understood.

POU-M2 is a homologue of vvl and regulates the expression of multiple genes in the silkworm. Here, we found that POU-M2 and JH synthase genes were expressed in BmE cells, and the overexpression of POU-M2 promoted JH synthase genes expression in BmE cells (figure 4), indicating BmE cells are applicable for the regulatory landscape in the CA of silkworm larvae.

As a key rate-limiting enzyme for JH synthesis, JHAMT is transcript-specific expressed in CA [41] and not expressed at all in other tissues. In contrast, POU-M2 is a ubiquitous factor expressed in different tissues and regulates the expression of many genes. POU-M2 is highly expressed in CA to promote JHAMT expression, but it is not a CA-specific gene-regulating JHAMT expression. We found POU-M2 was highly expressed in anterior/middle silk gland (electronic supplementary material, figure S1), implying a key role of POU-M2 in these tissues.

POU-M2 transcripts were continuously expressed from L3 to wandering stage, whereas JHAMT transcription was completely shut down after L5D3 (figure 3). However, the transcription of other JH synthases genes did not stop after L5D3 (figure 3). The transcriptional profiles of other JH synthases genes were similar to that of POU-M2. Our results showed POU-M2 was also involved in the expression of these genes (figures 4 and 5). Hence, we hypothesized that POU-M2 regulates the expression of JH synthase genes before L5D3, thus regulating JH synthesis, and still regulates the expression of JH synthase except for JHAMT after L5D3, but JH synthesis is aborted as JHAMT transcription is completely turned off.

The specific super-shift band did not appear after incubation with anti-POU-M2, the probe and nuclear protein (figure 7), possibly because the binding of anti-POU-M2 to POU-M2 blocks the binding site of DNA probe and POU-M2, causing the failure of the probe binding to POU-M2.

The conserved homeodomain and POU-specific domain are essential for POU factors. Any mutation in the DNA junction region of the POU domain abolishes the high-affinity and site-specific DNA binding of POU factors [15,49,50]. Here, the luciferase reporter assay showed the absence of either the POU domain or homeodomain resulted in a significant decrease in the luciferase activity (figure 8), indicating that the POU domain and homeodomain are essential for the activation of POU-M2 on JHAMT promoter.

Previous studies have shown POU factors influence the neuroendocrine system during puberty and early vertebrate development [17,20]. Figure 2 showed that the fluorescence signal of POU-M2 appeared in the nerve cord and the brain of silkworm larvae, implying POU-M2 likely plays a key role in the nerve cord and brain of the silkworm.

POU-M2 was highly expressed in the CA (electronic supplementary material, figure S1) where JH is synthesized and specifically localized in the nucleus of CA cells (figure 2). The overexpression of POU-M2 promoted the transcription of JH synthase genes (figure 4) and activated the activity of these gene promoters (figure 5). The expression profile of POU-M2 was similar to those of JH synthase genes in the CA (figure 3). Further, POU-M2 activated JHAMT promoter by directly binding to the key CREs (-207, -249 and -453) upstream of JHAMT promoter (figures 6–8). Taken together, these facts suggested POU-M2 promotes JH synthesis by activating the transcription of JH synthase genes in the CA. In particular, POU-M2 activated the transcription of JHAMT, a key enzyme for JH synthesis, by directly binding to -207, -249 and -453 CREs upstream of JHAMT promoter, thus regulating JH biosynthesis in the CA of the silkworm.

Silencing of vvl by RNAi influences the biosynthesis of JH and ecdysone in *T. castaneum* [36] and *O. fasciatus* [37]. Here, we demonstrated POU-M2 is highly expressed in the CA of the silkworm to promote JH biosynthesis by directly activating the transcription of JH synthase genes, which is a significant step forward in the transcriptional regulation of JH biosynthesis. A detailed study on JH titration *in vivo* is still needed to better understand the regulation of POU-M2 on JH biosynthesis. Also, Meng *et al*. found that POU-M2 directs ecdysteroid synthesis by modulating the transcription of phantom and spook [30]. Therefore, further work should be concerned with the dynamics of JH and ecdysone regulated by POU-M2, which determine the larval moulting and metamorphosis of insects. As a key hormonal regulator, POU-M2 may be involved in comprehensive physiological activities, and its role in insect development and metamorphosis remains to be fully elucidated.

In conclusion, we have shown that POU-M2 is highly expressed in the CA of silkworm larvae to promote the transcription of JH synthase genes. Especially, POU-M2 activates JHAMT transcription by directly binding to the key CREs -207, -249 and -453 upstream of JHAMT promoter. POU domain and homeodomain are vital for POU-M2 activation. Our study is of great significance towards a better understanding of JH biosynthesis and insect development.

## Data Availability

This article has no additional data.
